# The economic burden of the post-COVID-19 condition: Underestimated long-term consequences of neuropsychological deficits

**DOI:** 10.7189/jogh.13.03019

**Published:** 2023-05-05

**Authors:** Philippe Voruz, Frédéric Assal, Julie A Péron

**Affiliations:** 1 Clinical and Experimental Neuropsychology Laboratory, Faculty of Psychology, University of Geneva, Switzerland; 2 Neurology Department, Department of Clinical Neurosciences, Geneva University Hospitals, Switzerland; 3 Faculty of Medicine, University of Geneva, Switzerland

An increasing number of observational studies suggest that 10%-35% of people who contract coronavirus (COVID-19) may go on to experience persisting symptoms over many months [1]. The post-COVID-19 condition (long COVID) leads to functional impairment and low productivity at work [1]. A recent 2021 Swiss survey conducted by the Federal Social Insurance Office [2] found that 2.27% of new disability insurance claims were due to the post-COVID-19 condition, increasing to 2.50% in 2022 (as of November 2022). These data could be a first glance at a potentially substantial number of individuals suffering from post-COVID-19 conditions who are also off work and are not yet eligible for social security disability insurance. Interestingly, recent data estimated that one million individuals being out of work due to the post-COVID-19 condition would cost approximately US$50 billion annually in the USA only because of the resulting reduction in labour supply [3]. This estimation does not consider possible direct additional costs associated with the management of the pathology (eg, medical care, medication) [1]. For example, myalgic encephalomyelitis (a pathology often compared to post-COVID-19 condition) is estimated to cost US$9000 annually per patient in the USA [3]. Therefore, direct and indirect costs of the post-COVID-19 condition have been recently estimated to range from US$140 to US$600 billion annually [4]. Moreover, one of the most prevalent and disabling symptoms of the post-COVID-19 condition is “brain fog”, reflecting the persistence of neurocognitive symptoms such as confusion, forgetfulness, a lack of focus and mental clarity, and fatigue [1,5]. Those were recently categorised as neuropsychological post-COVID-19 conditions, with 30%-40% of patients suffering or complaining of such symptoms [5], requiring exhaustive neuropsychological assessments from neuropsychologists. The latter may have several subtypes, and some patients may be unaware of their cognitive deficits [6]. Fine-grained analyses are needed to identify specific neuropsychological deficits or phenotypes of the post-COVID-19 condition.

The condition has also raised interest among epidemiologists, but its potential burdens on society, the economy, and the healthcare system have yet to be fully quantified. Recently, a new form of interdisciplinary research has emerged aimed at quantifying the economic burden of cognitive disorders associated with several neurological conditions, including multiple sclerosis, Alzheimer’s disease, and traumatic brain injury [7,8]. Studies have highlighted a range of dissociated effects dependant on the nature of the cognitive disorders. For example, a deficit in attention or concentration may not have the same economic and societal consequences as a memory disorder. Research on multiple sclerosis, which can cost annually up to AUS$68 000 per patient in Australia, has demonstrated long-term cognitive deficits. In particular, memory and mental speed were more predictive of the economic burden than disease stage, as measured by the Expanded Disability Status Scale (EDSS) [7]. This suggests the primordial role of neuropsychological assessment in the evaluation and detection of risk factors that can lead to amplified direct and indirect costs and that could potentially be mitigated by cognitive rehabilitation interventions. Moreover, a recent European study showed that families with cognitive impairment require a 48% higher household income to achieve a normal living standard [8]. Therefore, the economic burden would not only be limited to direct and indirect costs for the patient, but also to their closer family circle, thus exacarbating the economic toxicity on society and highlighting the significant impact that cognitive disorders have on daily life. As recently demonstrated by our group (COVID-COG project, University of Geneva and University Hospitals of Geneva), patients with the post-COVID-19 condition have higher cumulative levels of memory, executive, visuospatial and logical reasoning deficits than we would expect to observe in the general population [5]. This is associated with altered brain structural [9] and connectivity patterns [10], suggesting that many of the patients included in the Geneva COVID-COG Cohort developed a moderate to severe neuropsychological syndrome [5], contributing to greater health care demands [1], needed sick leave, lower quality of life, [6] and various other costs. Considering that current studies mainly rely on self-reported data, a sizeable percentage of patients may remain undetected, as specific patterns of reduced brain connectivity could mean they are unaware of their neuropsychological deficits [6]. As previously demonstrated, infectious diseases could trigger neurodegenerative pathologies (eg, Alzheimer’s disease) or, as shown by molecular data, an acceleration of neurodegenerative molecular cascades in patients hospitalized with SARS-CoV-2 [11]. If we consider the impact of this potential phenotype of the post-COVID-19 condition and the potential increase in chronic neurological disease following infection on daily life, we can assume that the estimated economic burden of the post-COVID-19 condition would be even greater.

Thus, it is essential to estimate the economic burden of the post-COVID-19 condition according to its neuropsychological disorders and phenotypes, based on multidisciplinary approaches, and statistically validated simulation analyses, as had been done for other neurological pathologies. The implementation of such interdisciplinary studies for the neuropsychological post-COVID-19 condition could have effects on encouraging the search for the most prominent neuropsychological risk factors on direct and indirect costs (as it has already been done for multiple sclerosis), designing targeted rehabilitation programs (which are currently lacking) based on evidence-based medicine, and developing specific guidelines associating neuropsychological disorders and professional recovery. This would allow employers to reduce the impact of neuropsychological disorders on their employees' productivity and positively impact society by reducing the disability costs of these syndromes and the impact on the patients and their surroundings.

**Figure:**
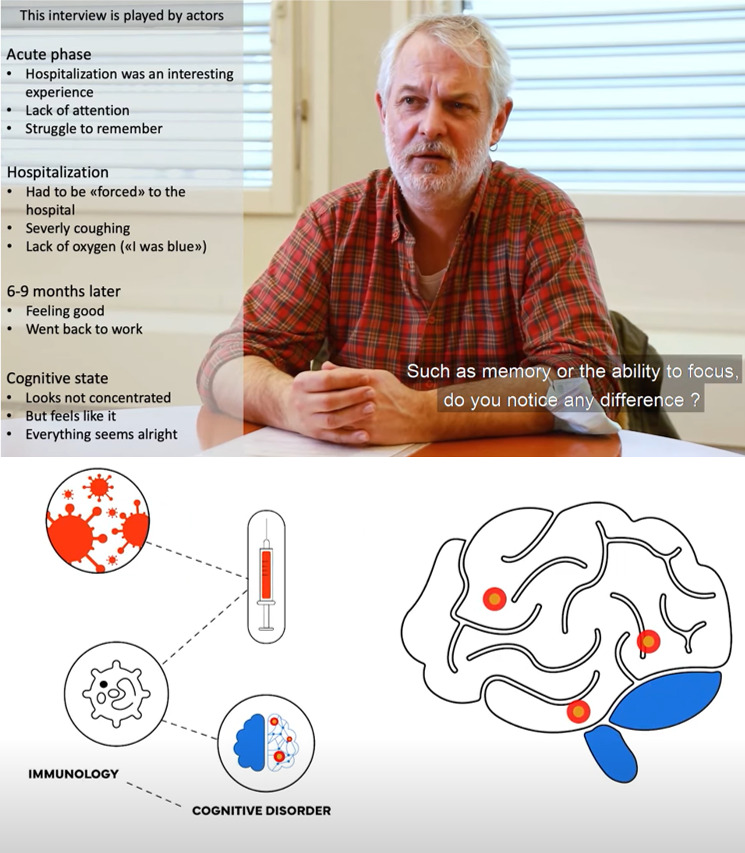
Photo: Anamnesis of a simulated patient suffering from anosognosia following a SARS-CoV-2 infection and schematic explaining potential association between immunological variables and long-term cognitive disorders following a SARS-CoV-2 infection. Source: Extracted from videos produced by the Clinical and Experimental Neuropsychology Laboratory (CENLab) in the framework of the COVID-COG project (NRP 78_Swiss National Science Fondation). Available: https://www.youtube.com/@nrp7854. © CENLab. Used with permission.

Finally, based on patient testimonials, a gap exists between empirical evidence of post-COVID-19 symptoms and the knowledge of health care professionals and economists. The scientific community and civil society must work together to develop effective communication channels based on empirical evidence to ensure clear and unbiased transmission of scientific results to all layers of society (e.g. patients and health care professionals, employers, economic entities, insurers) to mitigate the potential global impact of the neuropsychological post-COVID-19 condition.
